# Knowledge About Sounds—Context-Specific Meaning Differently Activates Cortical Hemispheres, Auditory Cortical Fields, and Layers in House Mice

**DOI:** 10.3389/fnins.2016.00098

**Published:** 2016-03-14

**Authors:** Diana B. Geissler, H. Sabine Schmidt, Günter Ehret

**Affiliations:** Institute of Neurobiology, University of UlmUlm, Germany

**Keywords:** c-FOS immunocytochemistry, context-specific auditory perception, left-hemisphere dominance, maternal cognition, maternal priming, plasticity of cortical activation, ultrasound perception

## Abstract

Activation of the auditory cortex (AC) by a given sound pattern is plastic, depending, in largely unknown ways, on the physiological state and the behavioral context of the receiving animal and on the receiver's experience with the sounds. Such plasticity can be inferred when house mouse mothers respond maternally to pup ultrasounds right after parturition and naïve females have to learn to respond. Here we use c-FOS immunocytochemistry to quantify highly activated neurons in the AC fields and layers of seven groups of mothers and naïve females who have different knowledge about and are differently motivated to respond to acoustic models of pup ultrasounds of different behavioral significance. Profiles of FOS-positive cells in the AC primary fields (AI, AAF), the ultrasonic field (UF), the secondary field (AII), and the dorsoposterior field (DP) suggest that activation reflects in AI, AAF, and UF the integration of sound properties with animal state-dependent factors, in the higher-order field AII the news value of a given sound in the behavioral context, and in the higher-order field DP the level of maternal motivation and, by left-hemisphere activation advantage, the recognition of the meaning of sounds in the given context. Anesthesia reduced activation in all fields, especially in cortical layers 2/3. Thus, plasticity in the AC is field-specific preparing different output of AC fields in the process of perception, recognition and responding to communication sounds. Further, the activation profiles of the auditory cortical fields suggest the differentiation between brains hormonally primed to know (mothers) and brains which acquired knowledge via implicit learning (naïve females). In this way, auditory cortical activation discriminates between instinctive (mothers) and learned (naïve females) cognition.

## Introduction

Mammals including humans can perceive acoustic messages in a given behavioral context as meaningful, if the heard sound patterns match the context, and sound and context are congruent with the physiological state of the individual. The evaluation of sounds in the auditory system leads to the representation of acoustic object properties in the auditory cortex (AC; Ehret, 1997; Nelken and Bar-Yosef, 2008; Kanwal and Ehret, 2011; Bathellier et al., 2012). The coding of acoustic patterns and objects in the AC is highly complex because information about the physiological state of the individual and non-auditory information about the behavioral context introduce great plasticity to neuronal responses. Neurons may change their activity with the acoustic scene and the pursued task (Fritz et al., 2003; Scheich et al., 2007; Atiani et al., 2009, 2014; Otazu et al., 2009; Chandrasekaran et al., 2010), including plasticity to changing vigilance and attention (Edeline et al., 2001; Bidet-Caulet et al., 2007; Fritz et al., 2007), and experience with and learning of sound patterns (Ohl and Scheich, 2005; Irvine, 2007; Scheich and Ohl, 2011; Weinberger, 2011; Lin et al., 2013). Further, hormones important in reproduction such as estrogen and oxytocin may introduce plasticity to auditory cortical processing (Banerjee and Liu, 2013; Elyada and Mizrahi, 2015; Marlin et al., 2015) by influencing the balance between excitation and inhibition in AC layers (Cohen and Mizrahi, 2015; Froemke, 2015; Marlin et al., 2015). Despite providing deep insight in the nature of plasticity of coding in the AC, the studies are not yet clear about the way of representation meaning of a given sound in the AC. This is especially true for the meaning of species-specific communication sounds and possible differences of representation between auditory cortical fields. We as human observers can only take the behavioral response of an animal as indicator for the perception of meaning. If the animal responds to a given sound of a conspecific or to an acoustically adequate model of the sound (not to an inadequate sound) with adaptive behavior (not with another type of behavior) both in an evolutionary sense and in relationship with a learned context, we can state the perception of the meaning of the sound. And since we observed some behavior, we can infer that the animal did not only perceive the meaning of the sound, but also was motivated to respond. Therefore, in addition to the representation of meaning, we can ask for neural correlations of motivated responses in the auditory cortex in case the experimental paradigm allows such responses. So far, studies measuring neuronal activation to sounds in the AC were restricted either to primary fields, and/or to anesthetized or head-fixed animals unable to adequately respond to the perceived sounds, and/or to sounds acoustically outside the species repertoire, and/or to sounds the significance of which was introduced via conditioning paradigms in more or less artificial laboratory settings.

Here, we use a well-characterized behavioral and perceptual paradigm in house mice in combination with the method of neuronal activation mapping via c-FOS immunocytochemistry (Sheng et al., 1990; Herrera and Robertson, 1996; Geissler and Ehret, 2004; Bourgeois et al., 2012; Geissler et al., 2013; Gaykema et al., 2014) to differentiate stimulus-specific, task-related (motivated vs. non-motivated vs. anesthetized), and experience- vs. hormone-primed neural activation in primary and higher-order fields of the AC. We ask how acoustic models of mouse pup ultrasound are activating the AC of female house mice when the sounds are meaningful or not meaningful in a maternal task. The meaning of the sounds is controlled both by the acoustic quality of the sounds (adequate vs. non-adequate sounds), the behavioral context of the animals, and by using animals which became maternally motivated in different ways, mothers through experience with the hormonal and sensory changes during pregnancy and giving birth (Rosenblatt et al., 1988; Scanlan et al., 2006; Brunton and Russell, 2008), virgin females through experience by co-caring for pups for 1 or 5 days (Ehret et al., 1987; Ehret and Koch, 1989; Stolzenberg and Rissman, 2011; Lin et al., 2013). A group of anesthetized virgin females with 5 days of pup-caring experience (PE) shows how the AC is activated by adequate sound when the animal is unable to actively listen and respond. Following up our previous FOS labeling studies of the AC (Fichtel and Ehret, 1999; Geissler and Ehret, 2004) and limbic system (Geissler et al., 2013) of the mouse in the context of maternal behavior, the following hypotheses are in the center of our present study: (1) As found before (Geissler and Ehret, 2004), the left-hemisphere AC may be activated over a larger area than the AC on the right side, independent of the perceived sounds. (2) Activation of primary fields (AI and AAF) of the AC may reflect acoustic properties of the sounds, not knowledge about sounds and their meaning. (3) Activation of the higher-order fields AII and DP may differ because of differences in the acquisition of maternal knowledge. This hypothesis specifies general plasticity in the AC in the course of becoming a mother (Elyada and Mizrahi, 2015) to higher-order fields. (4) Representation of acoustic meaning as defined above, and motivation to respond may be related to activation in the same higher-order field(s) in the AC. (5) If the ultrasonic field (UF) were part of the primary AC, its activation profile including that of supra- and infra-granular layers should correspond closely to the profiles in AI and AAF. (6) Activation of supra- and infra-granular layers in the fields of the AC may differ between animal groups independent of the perceived sounds but depending on the behavioral responses of the animals. This hypothesis refers to major differences in processing functions of these layers (e.g., Atencio et al., 2009). (7) Similar to human speech perception, we expect left-hemisphere dominant activation in the higher-order field (or fields) of the mouse AC which is (are) related to the perception of meaning.

Our results do not only show striking differences in the processing and representation of the meaning of sounds between the AC of the left and the right cortical hemispheres but also between primary and higher-order fields including supragranular and infragranular cortical layers. In addition, knowledge representation in the auditory cortical fields via hormonal priming appears to be different from that established via implicit learning.

## Materials and methods

### Animals

House mice (Mus musculus domesticus, outbred cross between the NMRI strain and feral mice, from our own breeding colony) aged 8–12 weeks were used. Two groups of primiparous mothers and five groups of virgin females (*n* = 7 in all groups) were involved. Animals were kept in plastic cages (26.5 × 20 × 14 cm) at 22°C and at a 12 h light-dark cycle (light on at 7 h). Food and water were available *ad libitum*. The experiments were carried out in accordance with the European Communities Council Directive (86/609/EEC) and were approved by the appropriate authority (Regierungspräsidium Tübingen, Germany).

### Experimental groups and acoustic stimulation

**Figure 1:**
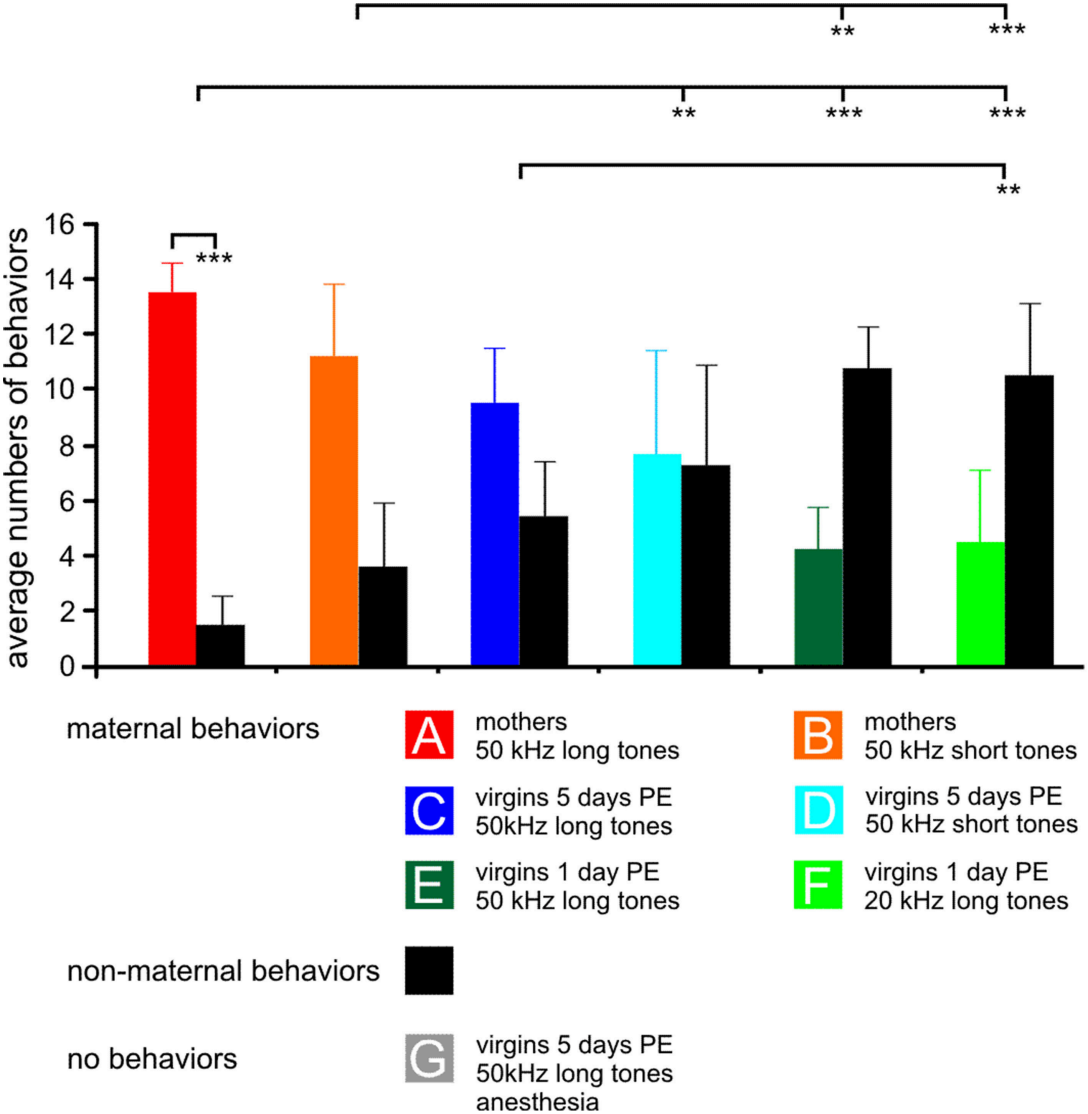
**The behavior of the animals in the experimental groups (A–G) during 15 min stimulation with the indicated sound for *c-Fos* expression**. For each group, 15 samples of behavior are divided in maternal (colored bars) and non-maternal (black bars) behaviors, respectively. The anesthetized animals could not show any behavior, so no bars are plotted. Means with standard deviations are indicated. PE, pup-caring experience. Statistically significant differences are *p* < 0.01 (^**^) or *p* < 0.001 (^***^). The indicated statistically significant differences between the groups refer to both maternal and non-maternal behaviors.

The seven experimental groups with their specific acoustic stimulation are indicated in Figure 1. Mothers (groups A, B) cared for their own pups for 5 days. Virgin females (groups C–F) were placed into the cage of a pregnant female (on day 18 of pregnancy) in order to experience delivery of the pups. Then, the virgins co-cared for the pups with the mothers for 1 day (groups E, F) or 5 days (groups C, D, G). Animals of groups A, C, E, G were stimulated with 50 kHz tone bursts of a duration of 60 ms including rise and fall times of 5 ms (= 50 kHz long tones). Animals of groups B and D were stimulated with 50 kHz bursts of a duration of 20 ms (2.5 ms rise and fall times included; = 50 kHz short tones) and group F was stimulated with 20 kHz bursts of a duration of 60 ms (5 ms rise and fall times included; = 20 kHz long tones). Animals of group G were anesthetized (see below) while being stimulated for *c-Fos* expression but otherwise they were identical to group C. 50 kHz long tones are acoustically adequate sounds, i.e., of high behavioral significance, to mothers (group A) and virgin females (group C and G) with 5 days of pup-caring experience (PE) for releasing a preferred approach to the sound source (Ehret et al., 1987; Ehret, 1992; Ehret and Buckenmaier, 1994). Anesthetized animals, of course, are unable to respond to the sounds. 50 kHz short tones and 20 kHz long tones are acoustically inadequate sounds (groups B, D, and F) and, therefore, of low behavioral significance for directing approach behavior to the sound source (Ehret et al., 1987; Ehret, 1992; Ehret and Buckenmaier, 1994). 50 kHz long tones, although acoustically adequate sounds to be perceived as highly behaviorally significant by mothers and virgin females with 5 days of PE, are sounds of low behavioral significance for releasing maternal behavior in virgin females with only 1 day of PE (group E; Ehret et al., 1987; Ehret and Buckenmaier, 1994).

Tones were generated with a digital frequency generator (Hewlett-Packard 33120A, USA) and shaped to tone burst series (3 bursts per second) of the desired burst duration (electronic switch, Uni-Konstanz-Elektronik, Germany). Sound pressure levels were adjusted to 80 dB for the 50 kHz tones and 66 dB for the 20 kHz tones (Kenwood RA 920 A, Japan; calibrated microphone, Bruel and Kjaer 4135 with preamplifier 2633 and measuring amplifier 2636) to provide about equal intensity of 60 dB above perceptual threshold for both frequencies (Ehret, 1974) in the central nest depression of a running board (length 110 cm, width 8 cm; Ehret and Haack, 1982; Geissler et al., 2013) which served as action space of the animals during the acoustic stimulation in a sound-proof and anechoic room under dim red light. The tones were fed through a custom-made voltage amplifier and power supply to two electrostatic loudspeakers (Machmerth et al., 1975) mounted 10 cm from the ends of the running board. At least 12 h before the acoustic stimulation, mothers with their pups or the mothers with their pups and the co-caring virgins were placed in the central nest depression of the running board, and the board was covered with a plastic hood. In that way, animals got accustomed to the room conditions and the running board. Before the acoustic stimulation the hood was removed. Mothers were removed before the acoustic stimulation of the virgin females. During the stimulation period of 15 min, tone bursts were constantly emitted by only one of the two loudspeakers at a time. Switching between the speakers was performed at intervals of 60—180 s. During the whole stimulation, some pups were taken from the nest area and placed on both sides of the running board. Thus, the experimental animals, while moving around on the running board, could find a pup and retrieve it back to the nest at any time. At the end of each minute of the 15 min tone stimulation the behavior of the animal was noted and classified as maternal (being actively engaged with a pup, licking or retrieving it or resting in the nest with pup contact) or non-maternal (doing something without active pup contact, i.e., moving around, sniffing, rearing, self-grooming, resting outside the nest). After the stimulation, the litter size was reduced to five pups in order to avoid production of wriggling calls by the pups in the nest (Geissler and Ehret, 2004), and the animals were kept additional 30 min in silence in the sound-proof room. The animals of group G were handled as the animals of group C until the mothers were removed from the running board. Then they were anesthetized with a single i.p. injection of a mixture of ketamine (120 mg/kg; Ketavet, Bayer) and xylazine (5 mg/kg; Rompun, Bayer) in 100 ml 0.9% NaCl. Usually within about 10 min the toe pinch reflex was gone and the anesthetized animals were placed in the nest area of the running board for sound stimulation. They remained there for the whole stimulation period and the 30 min observation in silence.

### Identification of the auditory cortex (AC)

**Figure 2:**
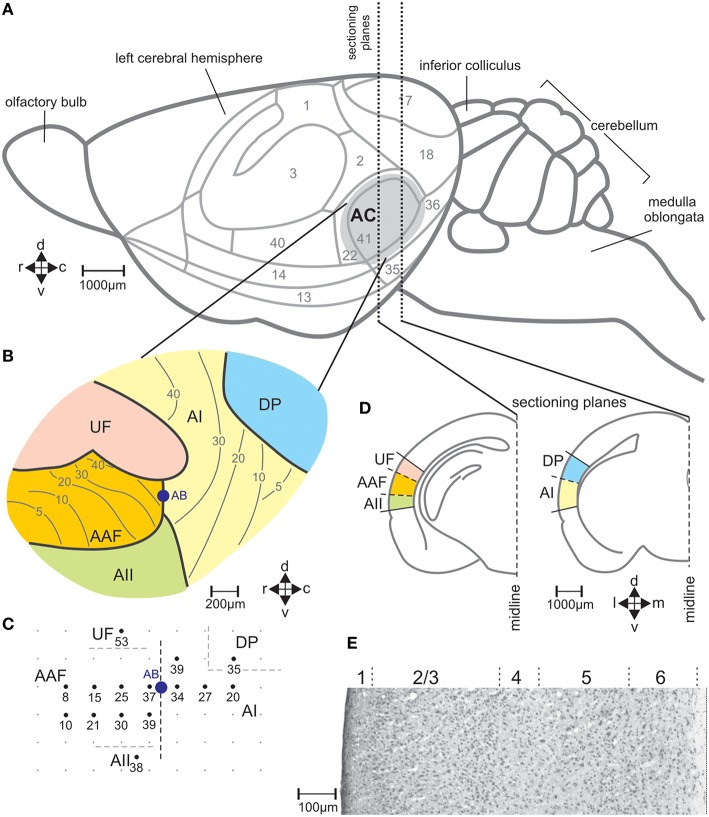
**The auditory cortical fields of the mouse brain**. **(A)** View on the left side of the brain with cytologically defined areas (Caviness, 1975) and the auditory cortex (AC) marked in gray. **(B)** Division of the AC in the fields AI, primary field; AAF, anterior field; UF, ultrasonic field; AII, secondary field; DP, dorsoposterior field, with the general tonotopic gradients in AI and AAF indicated by lines and the corresponding characteristic frequencies (kHz) (Stiebler et al., 1997). **(C)** Example of a map of electrophysiologically measured characteristic frequencies (kHz) of neurons at the indicated locations in the AC. AB shows the location at the border between AI and AAF where alcian blue was injected as reference in the brain sections for field assignment. **(D)** Two frontal sections through planes of the left-hemisphere AC with the positions of the indicated fields of the AC. **(E)** Sample section through AI stained with cresyl violet with indicated layers 1–6. c, caudal; d, dorsal; r, rostral; v, ventral.

In seven mice (one animal per experimental group A–G) the position of the left-side AC with its five fields (primary auditory field AI, anterior auditory field AAF, ultrasonic field UF, secondary auditory field AII, dorsoposterior field DP; Figures 2A,B) was defined with electrophysiological recordings. The auditory fields were distinguished by the response characteristics of the neurons and the frequency representation (tonotopy) within the fields (Stiebler et al., 1997; Joachimsthaler et al., 2014). Surgery and electrophysiology lasted about 4 h. They were exactly the same as previously described (Geissler and Ehret, 2004). Initial anesthesia by i.p. injection of 120 mg/kg ketamine, (Ketavet, Bayer) and 5 mg/kg xylazine (Rompun, Bayer) was maintained by 35 mg/kg ketamine and 1 mg/kg xylazine injected every 30–40 min. During surgery and recording, the body temperature was kept at 37°C (feedback-controlled heating pad; Harvard, Holliston, USA). The skin over the skull was incised at the midline, retracted, and the animal fixed in a head holder. Then, the cortical surface was exposed in the region of the AC. Extracellular responses from neuron clusters at a cortical depth of 400–500 μm were recorded with laquer-/glass-insulated tungsten microelectrodes (impedance 2–4 MΩ; Ciancone and Rebec, 1989). Recordings were done in a sound-proof, anechoic and electrically shielded room. Tone bursts (60 ms duration, 5 ms rise and fall time, 270 ms interburst interval) were generated (FG 1617, Voltcraft, Conrad Electronic, Germany; custom-made electronic switch), adjusted in sound pressure level (attenuator RA 920, Kenwood, Japan), amplified (PMA-1060, Denon, USA) and sent to a dynamic speaker (Dynaudio D28, Dynaudio, USA) and an electrostatic speaker (Machmerth et al., 1975) to cover the frequency range of 1-100 kHz with a flat ±5 dB sound field at the animal's ear. Neuronal activity was amplified, band-pass filtered (0.3–10 kHz; DAM 80, World Precision Instruments, USA) and fed to an audio monitor and oscilloscope. Characteristic frequencies (frequencies of lowest response thresholds) were determined audio-visually usually at 15 positions (about 200 μm distances between the recording points) of the AC (Figure 2C). The primary auditory fields (AI and AAF) were localized according to their tonotopic gradients, the other fields according to their relative position with regard to the primary fields (Figure 2C), and the differences of neural activity in these fields (non-tonotopic frequency representation, tone response latency, spontaneous bursting) compared to those in the primary fields (Stiebler et al., 1997). At the end of the mapping, the non-toxic permanent dye alcian-blue was injected at the border of AI and AAF (1% solution: pneumatic pico pump PV 830, World Precision Instruments). The positions of the recording sites and the alcian-blue injection site (reference point) were documented in scale drawings (Figure 2C). After the electrophysiological mapping, the cortical surface was covered with temporal muscle and the scalp was closed (Prolene monofil 0.7 metric, Ethicon, Germany) and an antibiotic salve applied to the suture (Nebacetin, Yamanouchi Pharma, Germany). Animals recovered from the surgery within 2 h and showed normal behavior. Behavioral tests with sound stimulation for *c-Fos* expression started 1 week after the surgery, at the earliest.

### *c-FOS* immunocytochemistry

The procedures were exactly the same as in Geissler and Ehret (2004) and Geissler et al. (2013). After the period in the sound-proof room, animals were killed by cervical dislocation, their brains quickly removed and frozen over liquid nitrogen. Frontal sections (30 μm) were made on a freezing microtome (HM 500OM, Microm, Germany), mounted on adhesion micro slides (HistoBond, Germany) and fixed with 4% paraformaldehyde. Then, the brains were processed for c-FOS immunocytochemistry using 0.2% Triton-X-100, 1% hydrogen peroxide and 2% normal goat serum all diluted in 0.1 M phosphate buffer (pH 7.4). Then the anti c-FOS rabbit primary antibody (Ab-5 PC38-100UL, Oncogene, USA or Calbiochem, Germany; diluted in 2% normal goat serum, 1:1000) and the horseradish peroxidase-conjugated secondary antibody (goat-anti-rabbit IgG/HRP PO 448, Dako, Germany; diluted in 0.1 M phosphate buffer, 1:200) were applied. After the treatment with paraformaldehyde, Triton-X-100, hydrogen peroxide and the primary antibody, slides were washed three times in 0.1 M phosphate buffer. The peroxidase reaction was done using diaminobenzidine and hydrogen peroxide, intensified by nickel chloride. The dehydrated sections on the slides were embedded in Entellan for microscopy.

### Data analysis

Analysis was done with a microscope (Axiophot, Zeiss, Germany) equipped with a CCD camera (1300B, VDS, Germany) and evaluation software (Lucia measurement 5.0). The left side AC of the seven electrophysiologically mapped mice was localized in the frontal sections by the alcian-blue mark (5x objective). To delimit the dorsoventral elongation of the auditory cortical fields in the sections, scale-edited models of the area of AII-AAF-UF and AI-DP (Figure 2D) were made on the basis of cytological structures (Caviness, 1975), and maps of positions and size relations of the AC fields (Stiebler et al., 1997). By superimposing the scale-edited model via a Camera lucida attached to the microscope over a frontal section to be evaluated, borders between the AC fields were determined in every section and FOS-positive cells assigned to a certain auditory field (Geissler and Ehret, 2004). FOS-positive cells were identified blind for the experimental group with a 10x objective as cells with dark-black round nuclei distinct from nuclei in shades of gray. The distinction between dark-black and gray staining was verified by computer-based evaluation of sample sections. The gray-values of the darkest pixels in the cell nuclei of the counted cells were measured and the value of the least intensely stained nucleus of these counted cells taken as threshold. With this threshold, all labeling above the threshold was automatically evaluated in the respective section again with the result of an objective identification of FOS-positive cells. There was less than average 1% deviation in counts of FOS-positive cells whether sections were evaluated visually or computer-based. Since we did not amplify the staining intensity (e.g., by using an avidin-biotin protocol) and counted only very black cell nuclei, we are confident that our cell counts recorded only those few neurons that were most strongly activated in animals of a given behavioral state by specific sounds in the specific behavioral context. These neurons may best indicate differences in stimulus-related and state-dependent activation of brain areas between the experimental groups of animals (Zuschratter et al., 1995; Geissler and Ehret, 2004).

**Figure 3:**
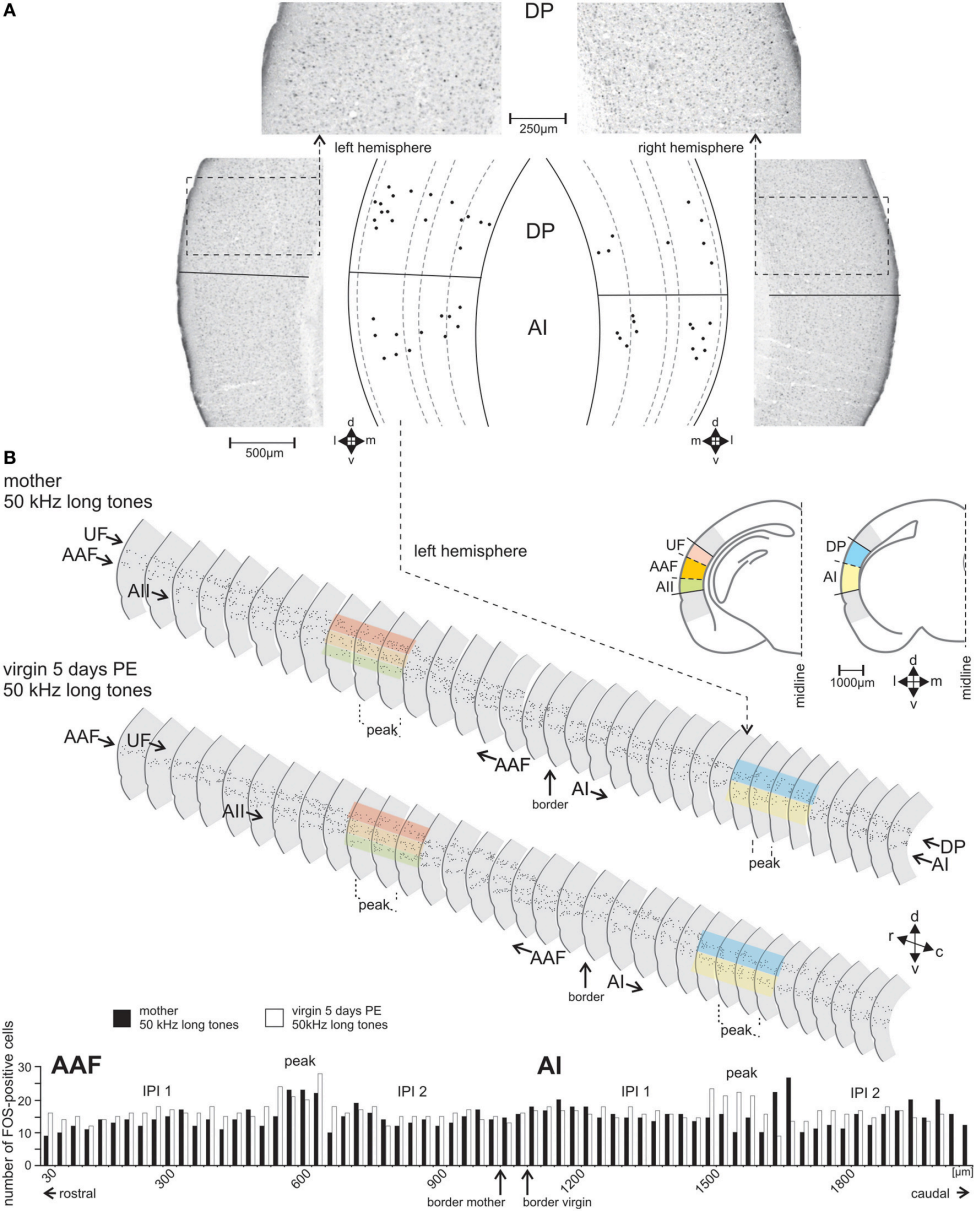
**The FOS-positive cells identified and counted in serial sections through the AC**. **(A)** Sample frontal sections through the left- and right-side AC of a group A mother in the area of the dorsoposterior (DP) and primary (AI) field with FOS-stained cells having nuclei in shades from black to gray. Cells counted as FOS-positive are indicated in the scale drawings as black dots at their positions relative to the cortical layers. **(B)** Scale drawings of the left-side AC of a group A mother and a group C virgin assembled in a serial reconstruction of the AC. Only every second section is shown. FOS-positive cells are indicated by dots. The colored bands over some sections show examples where the dorsoventral borders between the fields occurred. The histogram shows the numbers of FOS-positive cells in each section of AAF (anterior field) and AI of the mother (black bars) and the virgin (white bars). In the middle of both AI and AAF a peak in the number of FOS-positive cells (definition, see text) is visible with numbers higher than those in the inter-peak intervals (IPI 1, IPI 2) rostral and caudal of each peak. In the fields UF (ultrasonic field), AII (secondary field), and DP (dorsoposterior field) no peak areas occurred. c, caudal; d, dorsal; r, rostral; v, ventral.

With the stereotaxic relationships of the electrophysiological recording sites to the alcian-blue reference point at the AI-AAF border, this border was localized at average −2.92±0.08 mm posterior of the bregma point of the skull (Slotnik and Leonard, 1975; Paxinos and Franklin, 2001). In the region of the alcian-blue marked serial sections of the seven animals with electrophysiological mapping, we always found a change of the pattern of FOS-positive cells from three stripes of labeled cells rostrally to two stripes caudally (Figure 3B). As Figure 2C indicates, the three rostral stripes can be assigned to UF, AAF, and AII (from dorsal to ventral), the two caudal stripes to DP and AI. The same change of the pattern of FOS-positive cells was found at the equivalent position on the right hemisphere, which did not carry the alcian-blue mark. In this way, the AI-AAF border was not only defined by the position of the alcian-blue mark but also, and consistently, by the change from a pattern of three stripes of labeled cells in the cortical layers rostrally to two stripes caudally. Thus, with the relationship to the bregma-point and the relative positions of the auditory cortical fields to landmarks such as the rhinal fissure, the profile of the hippocampus, the shape of the corpus callosum and the piriform cortex, the AAF-AI border for a given animal and hemisphere without alcian-blue reference point was found and defined in the FOS labeled serial sections of this animal and hemisphere as shown in the two examples of Figure 3B. Once the AAF-AI border was defined, the beginnings of the fields UF, AII and DP were located in those sections from which stripes continued through the sections rostralward dorsal (UF) or ventral (AII) of AAF or caudalward dorsal (DP) of AI. The rostral or caudal borders of the respective fields were given by the sections in which these stripes vanished. In Figure 3B, the arrowheads related to AII and UF (UF only in the sections of the virgin) indicate the first rostral section without labeling.

FOS-positive cells were counted in every section of the tonotopically organized fields AI and AAF, and in the respective 10 central sections (300 μm) from the non-tonotopically organized fields UF, AII and DP. The counts were assigned to layers 2/3, 4, 5, or 6 of the cortex which can be distinguished by their cytoarchitecture (Frost and Caviness, 1980; Anderson et al., 2009; Broicher et al., 2010) both in sections stained with cresyl violet (standard staining protocol with sample sections of 50 μm thickness; Figure 2E) and in the sections stained with the immunocytochemical protocol for FOS (Figure 3A).

### Statistical analysis

Statistical tests were run with SigmaPlot (version 11.0) software. Since the data in our samples were normally distributed (Kolmogorov-Smirnov test) they were plotted as means with standard deviations and compared with parametric tests. In all tests, the given level of significance was α ≤ 0.01 (two-tailed) in order to identify only major differences. For statistical comparisons between measures from the left and right hemisphere of animals of a given experimental group or of other dependent data (e.g., maternal vs. non-maternal behaviors of a given experimental group; numbers of FOS-positive cells in a certain layer of a hemisphere of animals of a given experimental group vs. the average background counts of these animals), the paired *t*-test was used. The *t*-test was used to compare two sets of independent data. The one-way ANOVA followed by the Tukey-test (multiple comparisons of pairs) was used to test for differences in data between the experimental groups of animals (independent variable). In the text, the respective F and DF (residual) values are indicated in cases statistically significant differences were detected. In the figures concerning ANOVA results, the shown *p*-values indicate the results of the *post-hoc* Tukey-test.

## Results

### Behavioral significance of the sound stimuli

The behavioral data shown in Figure 1 are from animals of which results have been published in Geissler et al. (2013) supplemented by one additional animal each in groups A, B, E, and two additional animals in group C and D so that all these groups comprise seven animals, and by the new group G. Data from previous studies (Ehret et al., 1987; Ehret, 1992; Ehret and Buckenmaier, 1994; Lin et al., 2013) showed that stimuli such as 50 kHz long or short tones, or 20 kHz long tones were of different behavioral significance, i.e., of different meaning to the animals in the seven experimental groups: Natural ultrasounds of pups were adequately imitated by 50 kHz long tones being selectively attractive to groups A and C (Figure 1). 50 kHz short tones were less attractive than the 50 kHz long tones to mothers (group B) and 50 kHz short tones and 20 kHz long tones were rather unattractive to the virgins (groups D–F). Besides this different perception of the meaning of sounds, animals also expressed different degrees of maternal motivation visible in the rate of maternal behavior during the 15 min sound stimulation for *c-Fos* expression (Figure 1). Mothers were highly engaged in maternal behavior (almost exclusively pup-retrieval), especially when hearing the 50 kHz long tones (paired-*t*-test, *p* < 0.001). Virgins with 5 days PE were also, but less than the mothers, engaged in maternal behavior. Virgins with 1 day PE were maternal at a low rate, independent of the type of sound and at significantly lower rates than the mothers (ANOVA, DF = 36; maternal behaviors: *F* = 14.10, *p* < 0.001; non-maternal behaviors: *F* = 13.97, *p* < 0.001; both with the same results of the *post-hoc* Tukey-test, *p* < 0.01 or *p* < 0.001), and anesthetized virgins with 5 days PE (group G) were unable to show any behavior (Figure 1) although they heard an adequately attractive sound.

In summary, previous studies indicated that 50 kHz long tones were preferred by mothers and virgins with 5 days PE in order to release maternal behavior. Present and previous observations showed that maternal motivation was present in all experimental groups except the anesthetized group G, however, highest in mothers, lowest in virgins with 1 day PE, and intermediate in virgins with 5 days PE.

### Organization and size of the auditory cortical fields in both hemispheres

The position of the mouse AC on the temporal neocortex is shown in Figure 2A. It is composed of the tonotopic primary (AI) and anterior auditory field (AAF), and the non-tonotopic ultrasonic field (UF), the secondary field (AII), and dorsoposterior field (DP; Figures 2B,C; Stiebler et al., 1997; Joachimsthaler et al., 2014). Figure 2D shows these fields in frontal sections. The six cellular layers are shown in an example section from AI in Figure 2E. FOS-positive cells were seen in AC of both hemispheres and in each field in all 49 animals of the seven groups. Figures 3A,B illustrate the general FOS-labeling patterns. FOS-positive cells often appeared in bands through the cortical layers, clustered in layers 2/3, 5, and 6, and were present in all sections that could be assigned to the AC. Actually, the rostrocaudal extent of the AC was defined by the number of sections showing, without interruption, the banded pattern of FOS-positive cells (Figure 3B). This pattern was lost at the rostral and caudal borders where we found several sections without FOS-positive cells.

**Figure 4:**
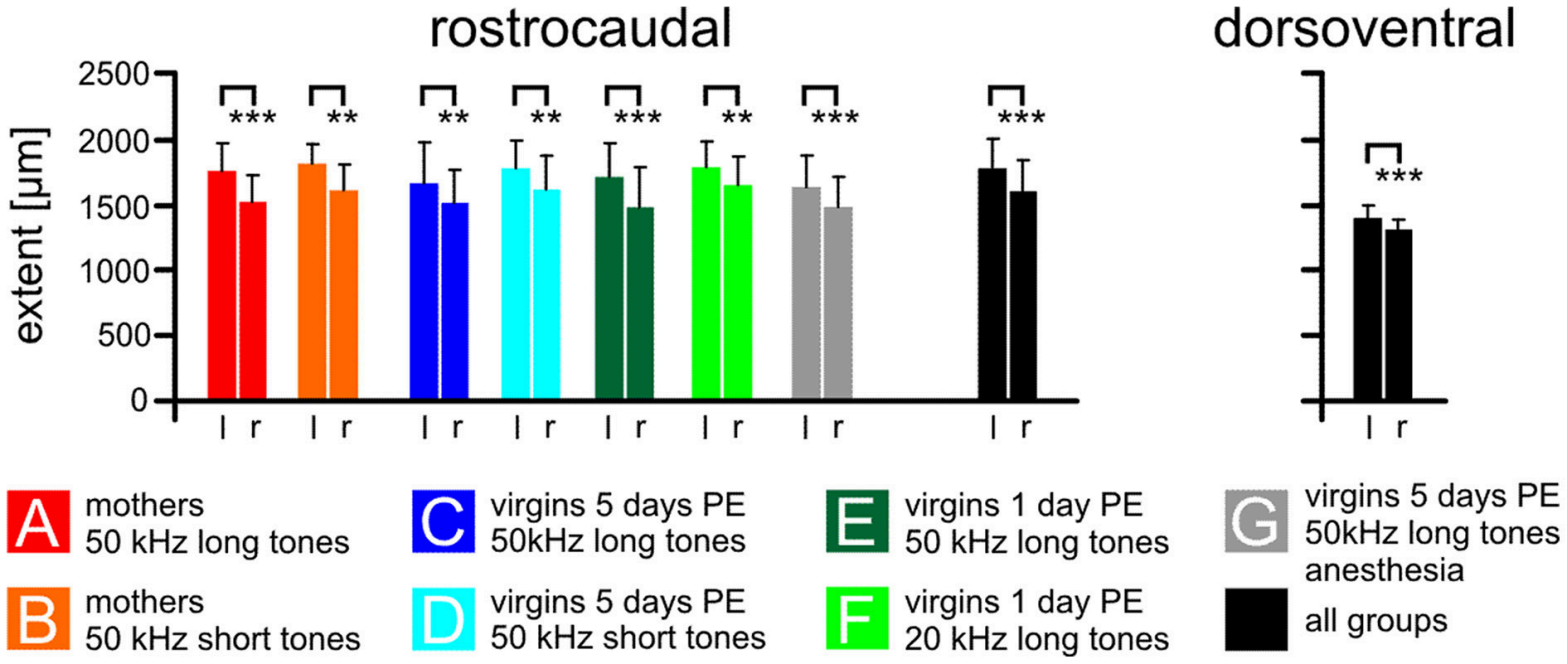
**The size of the AC on the left and right cortical hemisphere**. The average rostrocaudal extent by FOS-positive cells of the AC is significantly larger on the left compared to the right hemisphere in all experimental groups (A–G) and in all groups together. Also the dorsoventral extent of all groups together is larger on the left compared to the right side. Indicated are means and standard deviations. l, left hemisphere; r, right hemisphere. *p* < 0.01 (^**^); *p* < 0.001 (^***^).

The rostrocaudal extent of FOS-positive cells in the total AC did not differ among the experimental groups (ANOVA, *p* > 0.2) but was significantly larger in the left compared to the right hemisphere (paired *t*-test, *p* < 0.01 or 0.001; Figure 4). The mean rostrocaudal extent of the AC calculated from all animals in all groups was 1767 μm on the left and 1590 μm on the right hemisphere, leading to a highly significant difference (paired *t*-test, *p* < 0.001). The dorsoventral extent of the AC was determined about 500 μm caudal of the AI-AAF border. FOS-positive cells were also found over a larger extent in the left compared to the right hemisphere (Figure 4; mean left side: 1387 μm; mean right side: 1300 μm; paired *t*-test, *p* < 0.001). Statistically significant differences between the experimental groups did not occur (ANOVA, *p* > 0.2).

In summary, the functional area of the AC, as demonstrated by the strongly activated FOS-positive cells, was larger on the left compared to the right hemisphere.

### Quantification of FOS-positive cells in the primary fields AI, AAF, and in UF

**Figure 5:**
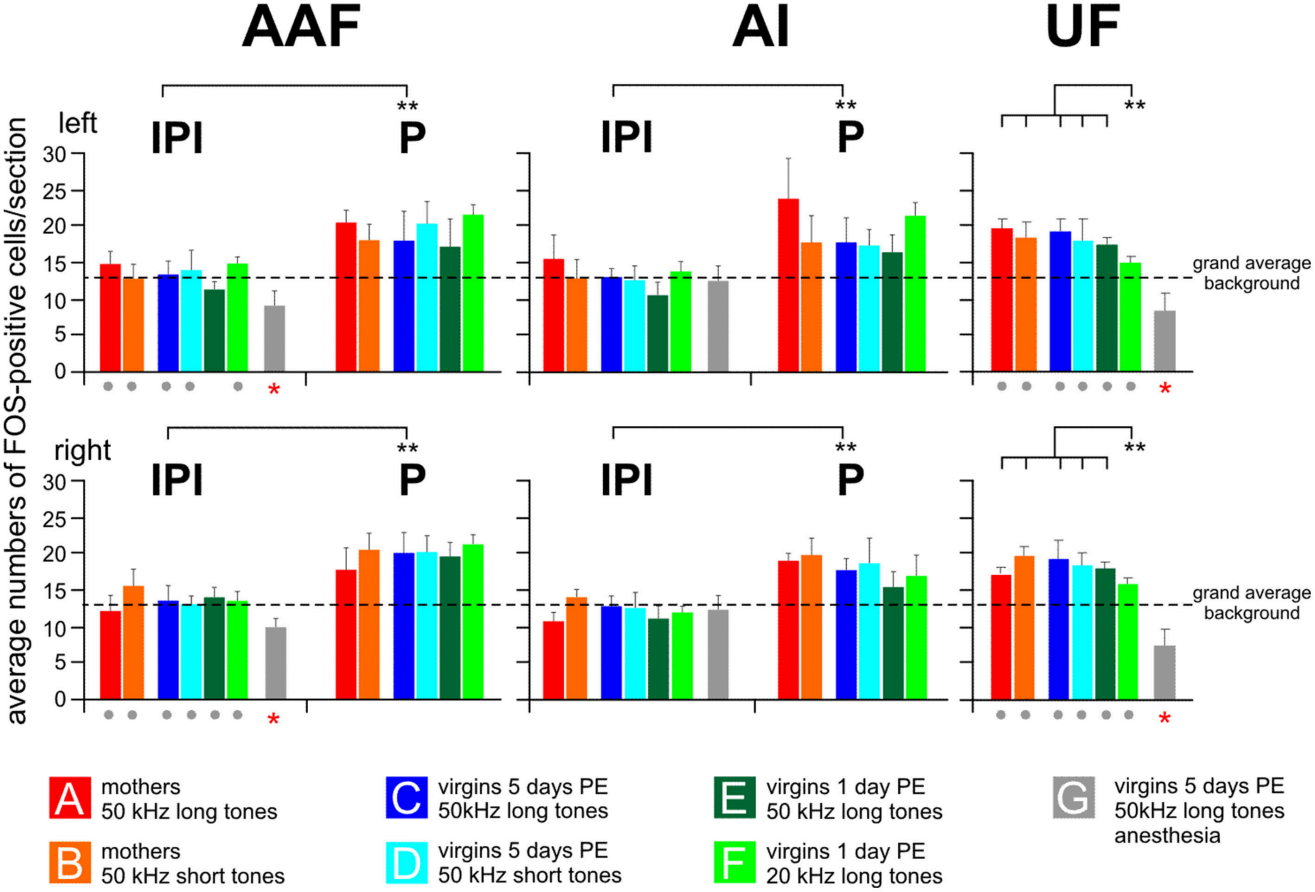
**Numbers of FOS-positive cells in the peak (P) and inter-peak (IPI) intervals of the primary (AI) and anterior (AAF) fields and in the ultrasonic field (UF)**. Means with standard deviations are shown for all experimental groups (A–G) separately for the left and the right hemisphere. The anesthetized animals (group G) did not show a peak area, neither in AI nor in AAF. Their cell counts were significantly lower (*p* < 0.01 at least) in AAF and UF of both hemispheres than the grand average background (red stars below the gray bars) and significantly lower than the cell counts for the awake animals in all the other groups (gray dots below the colored bars of the respective groups). The left and right-hemisphere IPI counts from AI and AAF of groups A–F were all significantly (*p* < 0.01) smaller than the P counts of the respective hemispheres and groups. This is indicated by the bars with ^**^ between IPI and P. In the UF, the number of FOS-positive cells in response to 20 kHz tones (group F) was significantly smaller (*p* < 0.01) than the numbers in all other groups (A–E) of awake animals hearing 50 kHz tones.

The section by section analysis of the FOS-positive cells showed that all awake animals in groups A–F but not the anesthetized animals of group G had in AI and AAF of both hemispheres one area of 2–4 adjacent sections with an increased number of 4–10 FOS-positive cells compared to the maximum number of labeled cells in any of the other sections of the given hemisphere of an animal (Figure 3B). Based on the number of 30 μm thick sections in the peaks concerning AAF and AI of all the animals, the mean width of this peak in AAF and AI was 82 ± 22 μm (almost three sections) with no differences between the hemispheres (paired *t*-test, *p* > 0.2) and the animals in the groups A–F (ANOVA, *p* > 0.2). This peak (P) occurred in AI and AAF of group A–F animals at an average rostrocaudal position of 47% of the total rostrocaudal extent of AI and AAF, respectively, away from the AAF-AI border (Figure 3B). Thus, both AI and AAF of the awake animals had a peak area and two areas rostral and caudal, respectively, of the peak which we named inter-peak intervals (IPIs). Since the average counts of FOS-positive cells in rostral and caudal IPIs of AI and AAF did not differ in any animal (paired *t*-test, *p* > 0.1), we calculated the average values from these IPIs in AI and AAF separately for each group and hemisphere of the awake animals and plotted the average IPI counts/section together with the respective average peak counts/section in Figure 5. As consequence of the definition of peaks and IPIs in the individuals, the average numbers of FOS-positive cells in the peak of AI and AAF of both hemispheres of groups A–F were significantly higher than in the corresponding IPI (paired *t*-test for each hemisphere and experimental group, *p* < 0.01, or *p* < 0.001). Figure 5 shows rather similar IPI counts in AI and AAF for both hemispheres and across all groups of awake animals. Actually, we did not find significant differences between the experimental groups (ANOVA, *p* > 0.2) and, therefore, averaged the IPI counts from AI and AAF of both hemispheres and all awake animals (groups A–F) with the resulting grand average background (13.5 FOS-positive cells/section) for the awake AI and AAF (dashed horizontal line in Figure 5).

The anesthetized animals (group G) did not show peak areas in AI or AAF. Therefore, all counts of FOS-positive cells from this group were taken as IPI or background. The average values for each hemisphere AI and AAF are plotted in Figure 5. The average AI IPIs of group G did not differ from the IPIs of the awake animals (ANOVA, *p* > 0.05) and were very similar to the grand average background of groups A–F (ANOVA, *p* > 0.1). The average AAF IPIs of group G were in both hemispheres significantly smaller (ANOVA, DF = 42; left: *F* = 8.82, *p* < 0.001; right: *F* = 7.50, *p* < 0.001; with *post-hoc* Tukey-test, *p* < 0.01 for both sides) than the IPIs of the awake animals, smaller than the AI IPIs of group G (paired *t*-test, *p* < 0.001), and smaller than the grand average background of groups A–F (ANOVA, DF = 42; left: *F* = 11.50, *p* < 0.001; right: *F* = 9.40, *p* < 0.001; with *post-hoc* Tukey-test, *p* < 0.001 for both sides; Figure 5).

The ultrasonic field (UF) had no peaks in the number of FOS-positive cells along the rostrocaudal extent in any hemisphere of all 49 animals. Therefore, we calculated the mean number of FOS-positive cells from the 10 evaluated sections separately for both hemispheres of each animal. Then, we averaged these numbers over all animals within an experimental group and plotted these cell counts/section in Figure 5. Mothers and awake virgins hearing the 50 kHz tones (long and short, groups A–E) showed about the same numbers of FOS-positive cells in UF (ANOVA, *p* > 0.2) without differences between the hemispheres (paired *t*-test, *p* > 0.1). These numbers did not differ from the peak values neither from AI or AAF (paired *t*-test, *p* > 0.1) in the respective groups but were significantly higher than the respective values in the IPIs (paired *t*-test, *p* < 0.01). Virgin females hearing the 20 kHz tones (group F) had smaller numbers of FOS-positive cells in UF than the awake animals in the other groups (ANOVA, *DF* = 36; left: *F* = 9.96, *p* < 0.001; right: *F* = 4.45, *p* < 0.01; with *post-hoc* Tukey-test, *p* < 0.01). Further, the UF counts of group F animals were very similar to their IPI counts both for AI and AAF (paired *t*-test, *p* > 0.1) and significantly smaller than the AI (ANOVA, *DF* = 36; left: *F* = 5.34, *p* < 0.001; right: 6.74, *p* < 0.001; with *post-hoc* Tukey-test, *p* < 0.01 for both sides) and AAF (ANOVA, *DF* = 36; left: *F* = 3.65, *p* < 0.01; right: 4.8, *p* < 0.01; with *post-hoc* Tukey-test, *p* < 0.01 for both sides) peak counts of the awake animals of groups A–E. The anesthetized females of group G had significantly smaller numbers of FOS-positive cells in UF than the awake animals in all the other groups (ANOVA, *DF* = 42; left: *F* = 11.41, *p* < 0.001; right: *F* = 6.48, *p* < 0.001; with *post-hoc* Tukey-test, *p* < 0.01 for both sides; Figure 5). The group G values in UF were also smaller than the grand average background from the awake animals of the other groups (A–F) (ANOVA, *DF* = 42; left: *F* = 12.69, *p* < 0.001; right: *F* = 20.98, *p* < 0.001; with *post-hoc* Tukey-test, *p* < 0.001 for both sides).

In summary, 50 kHz tones activated the UF significantly more than 20 kHz tones, however only in awake animals. Unexpectedly, in awake animals 50 kHz tones led to a local peak of activation above the general background in the middle of both AI and AAF. Compared to awake and behaving animals, anesthesia eliminated the peak activation in AI and AAF and significantly reduced the background activation in AAF and UF.

### Quantification of FOS-positive cells in the layers of AI, AAF, and UF

**Figure 6:**
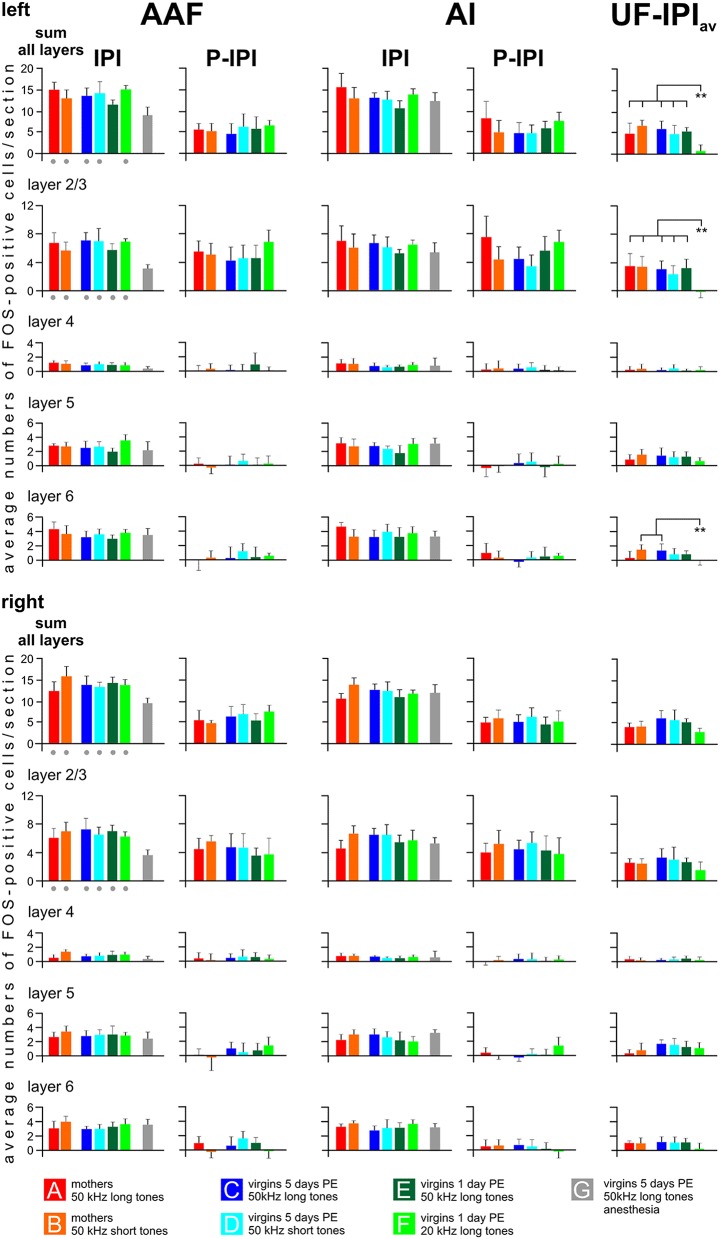
**Distribution of FOS-positive cells in the primary (AI), anterior (AAF), and ultrasonic (UF) fields in cortical layers**. Data for AAF and AI are shown separately for all experimental groups and both hemispheres as values (means with standard deviations) of the inter-peak intervals (IPI) and the peak after subtraction of the respective IPI value (P—IPI). The labeling peaks in AAF and AI clearly relate only to layers 2/3. The values for UF indicate the difference UF—IPI_av_, whereby IPI_av_ is the average IPI value from the AI and AAF of the respective groups. Labeling above the background is found in UF in layers 2/3, 5, and 6. IPI values of anesthetized animals (group G) are significantly smaller (*p* < 0.01) in the sum of all layers and in layers 2/3 of AAF than the respective values of all other groups (gray dots below the colored bars). The values UF—IPI_av_ of group F virgins are significantly smaller (*p* < 0.01) than the values of the indicated other groups. The anesthetized animals of group G did not show labeling peaks in AAF and AI and, in UF, no labeling above the background. Therefore, there are no gray group G bars in the P—IPI and the UF—IPI_av_ columns. *p* < 0.01 (^**^).

Figure 6 shows how the cortical layers in AI, AAF, and UF contributed to establish the general activation patterns. The IPI values for both AI and AAF hemispheres were obtained by taking the average IPI values per layer and the sum of all layers of each animal to calculate the respective group average. In order to find out how the excess labeling at the P area in AI and AAF of awake animals distributed across the layers, the average IPI value of each animal was subtracted from its average peak value separately for each layer and the sum of all layers, and this difference (P—IPI) was averaged across all animals of a given group. Similarly, the average IPI value concerning both AI and AAF of each awake animal (IPI_av_) was subtracted from its average UF value separately for each layer and the sum of all layers, and this difference (UF—IPI_av_) was averaged across all animals of a given group. Since the anesthetized animals of group G did not show a P area in AI and AAF and only reduced labeling below the grand average background in UF (Figure 5), their labeling in AI and AAF was counted as IPI and the respective average values for the layers and the sum of all layers were plotted in Figure 6.

**Figure 7:**
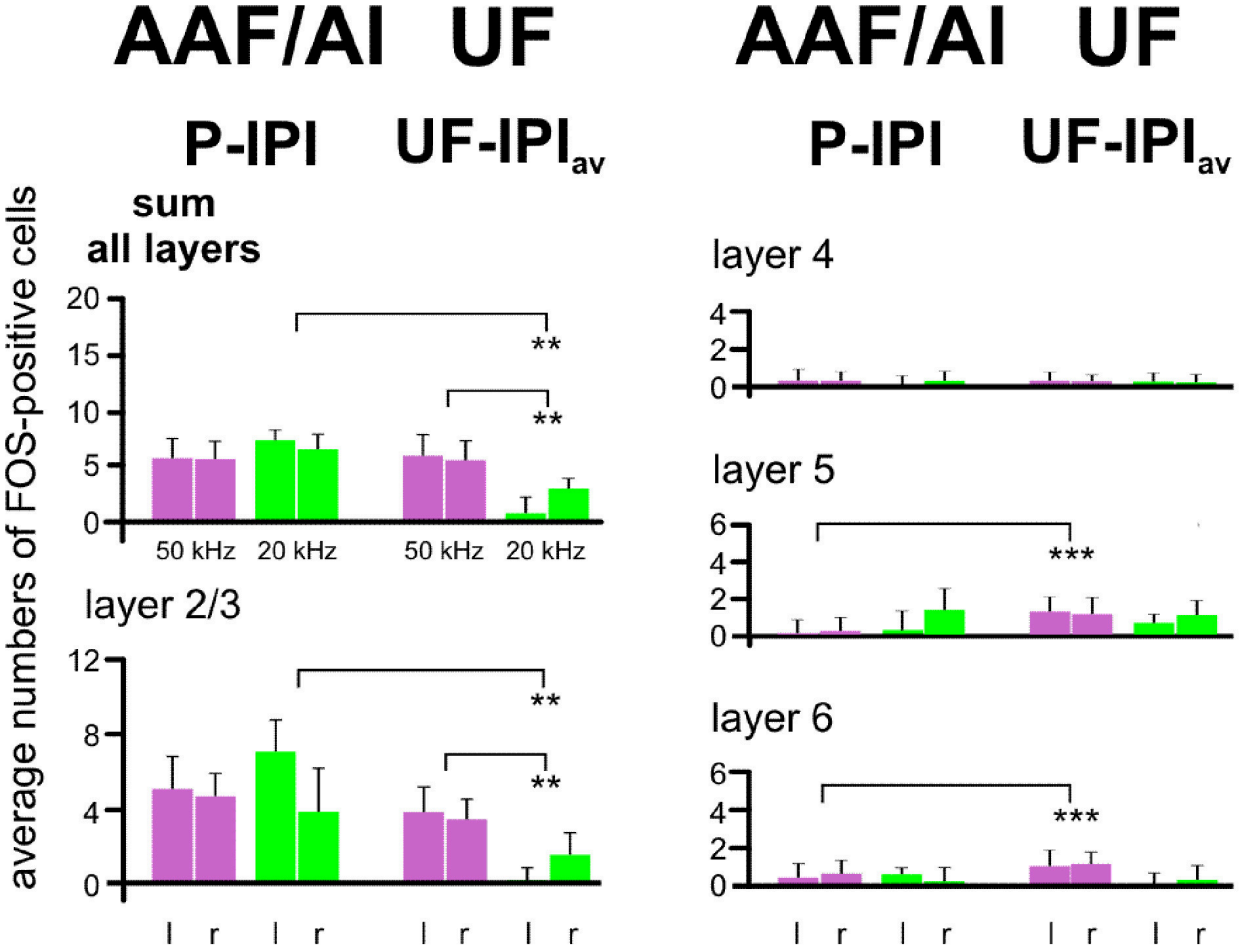
**Comparison of activation by 50 and 20 kHz tones in the primary (AI), anterior (AAF), and ultrasonic (UF) fields**. In this figure all data from AI, AAF, and UF of all experimental animals hearing 50 kHz tones (groups A–E) have been combined, leading to one average value for the inter-peak (IPI) and one for the peak (P) intervals in AAF and AI and one for the UF. The numbers of FOS-positive cells in the IPIs have been subtracted from the numbers in the peaks (AAF/AI), and the numbers in the average inter-peak intervals (IPI_av_, calculated from IPIs in AAF and AI) have been subtracted from the UF values (UF—IPI_av_) in order to create the plotted means with standard deviations. These average data from the animals in groups A–E are compared with the data of the animals hearing the 20 kHz tones (group F). The comparisons show that 20 kHz activation in AAF/AI (sum of all layers and layers 2/3), especially in the left hemisphere, is significantly stronger (*p* < 0.01) than in UF. Conversely activation by 50 kHz in UF (sum of all layers and layers 2/3) is significantly stronger (*p* < 0.01) than activation by 20 kHz. In addition, layers 5 and 6 are significantly more activated (*p* < 0.001) by 50 kHz tones in UF than in AAF/AI. l, left hemisphere; r, right hemisphere. *p* < 0.01 (^**^), *p* < 0.001 (^***^).

The results of the layer-related labeling patterns (Figure 6) show: (a) The background in the IPI areas of the primary fields (AI, AAF) continued through all layers with very similar numbers of FOS-positive cells in a given layer in both hemispheres and all groups with awake animals (A–F). Statistical analyses did not reveal significant differences in the numbers of FOS-positive cells for the sum over all layers or within a given layer (2/3, 4, 5, and 6) between the A–F groups (ANOVA, *p* > 0.1). There was no difference of IPI labeling in AI (both hemispheres) between the awake (A–F) and the anesthetized (G) animals (ANOVA, *p* > 0.05). Significant differences occurred, however, in AAF where group G animals had lower values in the sum of layers (ANOVA; *DF* = 42; left: *F* = 8.82, *p* < 0.001; right: *F* = 7.50, *p* < 0.001; with *post-hoc* Tukey-test, *p* < 0.01 for both sides) and in layer 2/3 (ANOVA; *DF* = 42; left: *F* = 10.60, *p* < 0.001; right: *F* = 8.41, *p* < 0.001; with *post-hoc* Tukey-test, *p* < 0.01 for both sides; Figure 6). (b) The P area in AI and AAF (both hemispheres) was defined by excess labeling above the background only in layer 2/3, again without significant differences between the groups (ANOVA, *p* > 0.1). The average cell counts above the respective backgrounds in the layers 4, 5, and 6 were all close to zero (Figure 6). (c) The number of labeled cells above the average background in UF (UF—IPI_av_) when the animals heard 50 kHz tones (groups A–E) did not only concern layer 2/3 but also layers 5 and 6. Generally larger numbers of FOS-positive cells were counted in layers 5 and 6 in the UF (UF—IPI_av_) compared to the numbers in the respective layers of the peak areas in AI and AAF (P—IPI; see Figure 5). These differences became statistically significant (*t*-test, *p* < 0.001) when the respective values from all animals of groups A-E were combined as shown in Figure 7. This figure also shows that stimulation with 20 kHz tones (group F) led to significantly larger numbers of FOS-positive cells (*t*-test, *p* < 0.01) in the sum of layers and in layer 2/3 in the P areas of AI/AAF compared to UF. In addition, 20 kHz stimulation in the experimental group F led to significantly smaller numbers of FOS-positive cells (*t*-test, *p* < 0.01) in the sum of layers and in layer 2/3 in UF compared to 50 kHz stimulation in the other groups (Figure 7). Hemisphere differences in these comparisons of labeling in UF, and UF with AAF/AI did not occur (paired *t*-test, *p* > 0.05, in most cases *p* > 0.1).

In summary, ultrasounds (50 kHz tones) activated cortical layers 2/3, 5, and 6 in the UF of all awake animals irrespective of the meaning of the sounds to the listening animals. The peak of labeling in AI and AAF in all groups of awake animals was due to increased activation only in layers 2/3. 20 kHz tones activated layers 2/3 more in AI/AAF than in UF, but did not lead to a significant number of labeled cells above background in layers 4, 5, and 6 of AI/AAF. Anesthesia significantly reduced labeling in all layers of UF, but only in layer 2/3 of AAF.

### Quantification of FOS-positive cells in the layers of the non-primary fields AII and DP

**Figure 8:**
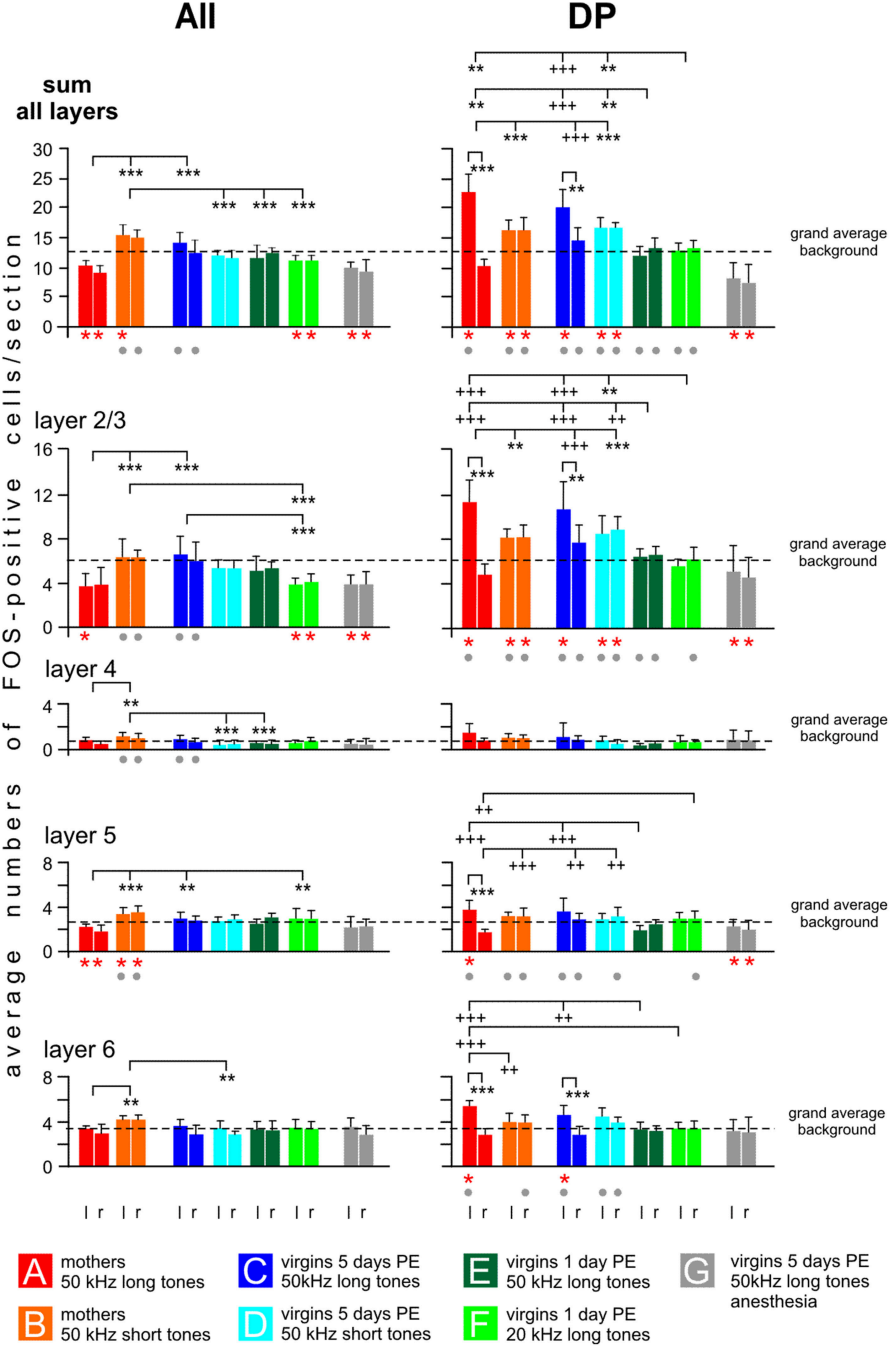
**Distribution of FOS-positive cells in the cortical layers of the secondary (AII) and dorsoposterior (DP) fields of the AC**. The plotted values (means with standard deviations) relate to the left (l) and right (r) hemisphere and to cortical layers 2/3, 4, 5, 6 and to the sum of all layers. Significant differences (*p* < 0.01, at least) between the grand average background of a group (calculation, see text) and the plotted group values are indicated by red stars below the respective group bars. Significant differences (*p* < 0.01, at least) between values of the anesthetized animals (group G) and any values of the others groups are indicated by gray dots below the bars of the group of non-anesthetized animals. Significant differences between values of groups or between the left and right hemisphere of a given group are indicated above the histograms. Stars (^**^ or ^***^) are used in cases when the inter-group differences relate to both hemispheres, plus signs (++ or +++) are used when the inter-group differences relate only to one hemisphere pointed at by the vertical dashes. *p* < 0.01 (^**^ or ++); *p* < 0.001 (^***^ or +++). For further explanations, see text.

Counts of FOS-positive cells in the non-primary fields AII and DP are shown in Figure 8 as the sum for all layers and specified for layers 2/3, 4, 5, and 6. In these fields, we did not see a local peak of labeling as in AI and AAF in any group. Therefore, as for UF, the numbers of FOS-positive cells for each animal were obtained by averaging the cell counts across the 10 evaluated sections separately for both hemispheres, the layers and the sum of the layers. These values of the individuals were then averaged across the animals of each group to be shown in Figure 8. We also indicated the level of the grand average background as calculated from the IPIs of AI and AAF of awake animals in order to demonstrate not only differences in activation between the experimental groups but also group-specific increases or decreases in activation relating to the common general level of awake animals.

In AII, possible left-right differences in the numbers of FOS-positive cells were analyzed in each experimental group separately for the sum of all layers and each layer and no statistical differences were obtained (paired *t*-test, *p* > 0.1 in each case). In contrast to the primary fields, 50 kHz tones activated AII differently depending on who was listening (ANOVA over all groups, *DF* = 42, *p* < 0.01 or *p* < 0.001; sum of all layers left: *F* = 16.86, right: *F* = 21.09; layer 2/3 left: *F* = 8.67, right: *F* = 8.27; layer 4 left: *F* = 8.64, right: *F* = 5.63; layer 5 left: *F* = 3.28, right: *F* = 7.14; layer 6 left: *F* = 3.74, right: *F* = 3.94). The most striking group characteristics (Figure 8): The highest counts of FOS-positive cells were found in mothers hearing the 50 kHz short tones (group B), both with regard to the sum in all layers and to the numbers in the individual layers. This high activation was especially prominent and significant throughout all cortical layers when compared with group A mothers hearing the 50 kHz long tones (Figure 8; separately for the layers with *post-hoc* Tukey-test, *p* < 0.01 or *p* < 0.001). Also the virgins with 5 days PE hearing the 50 kHz short tones (group D) and the virgins with 1 day PE hearing the 50 kHz long tones (group E) had significantly smaller numbers of FOS-positive cells than group B mothers (Figure 8; statistically significant for the sum in all layers and some individual layers, *post-hoc* Tukey-test, *p* < 0.01 or *p* < 0.001). The smallest numbers of FOS-positive cells in AII occurred in the mothers hearing the 50 kHz long tones (group A), the anesthetized virgins hearing the same sounds (group G), and the awake virgins hearing the 20 kHz tones (group F). Their numbers were even below the grand average background, significant for the sum of all layers and layer 2/3 and 5, the latter only in group A (paired *t*-test, *p* < 0.001 in each case).

In DP, hemisphere differences were noted for mothers and virgins with 5 days of PE hearing the 50 kHz long tones (groups A, C), i.e., there were significantly higher counts of FOS-positive cells in the left than in the right hemisphere for the sum of all layers, and for all individual layers of the mothers and layers 2/3 and 6 of the virgins (Figure 8; paired *t*-test, *p* < 0.01 or *p* < 0.001). Hemisphere differences were not present in the other groups (B, D–G). In general (ANOVA over all groups, *DF* = 42, *p* < 0.01 or *p* < 0.001; sum all layers left: *F* = 28.99, right: *F* = 26.66; layer 2/3 left: *F* = 24.63, right: *F* = 23.65; layer 5 left: *F* = 10.04, right: *F* = 10.2; layer 6 left: *F* = 9.3, right: *F* = 7.52), mothers and virgins with 5 days of PE (groups A–D) had significantly higher counts of FOS-positive cells than virgins with only 1 day of PE (groups E, F) and anesthetized animals (group G), irrespective of the sound they were stimulated with (*post-hoc* Tukey test, *p* < 0.01 or *p* < 0.001). This difference was present for the sum of labeled cells in all layers and layer 2/3, and for layers 5 and 6 of the left hemisphere (Figure 8). Virgins of groups E and F had about the same number of FOS-positive cells in DP of both hemispheres as the grand average background labeling (in sum and in all layers; Figure 8). Groups A–D (A and C only in the left hemisphere) had higher counts of labeled cells compared to the grand average background, significant (paired *t*-test, *p* < 0.01) in the sum of all layers and in layer 2/3 (in groups A and C also in layer 6, in group A also in layer 5). Anesthetized virgins (group G) had smaller numbers of FOS-positive cells in both hemispheres of DP (sum of all layers, layers 2/3 and 5) compared to the grand average background (*t*-test, *p* < 0.01 or *p* < 0.001).

In summary, activation of AII showed major differences between mothers (groups A, B) and virgin females (groups C–F), i.e., the processing of the different ultrasound models led to a large difference in activation of AII of the mothers but not of the virgins. The AII of anesthetized virgins was less activated, mainly in layers 2/3 and 5, compared to awake virgins hearing the same sounds. Activation of DP was especially high in the left brain hemisphere of group A and C animals. In addition, DP (at least of the left hemisphere) was highly activated in animals with high or medium maternal motivation (groups A–D; Figure 1), and activated only at the background level in animals of low maternal motivation (groups E, F) or even below the average background in anesthetized animals (group G). The group-specific activation patterns were often visible through all cortical layers.

## Discussion

### The message of FOS labeled cells about general activation in the AC

Without sound stimulus in the maternal context, there was little or no strong FOS labeling in the AC of the mouse (Fichtel and Ehret, 1999; Geissler and Ehret, 2004) or gerbil (Zuschratter et al., 1995), i.e., hearing sounds is one condition for highly activated cells to occur in the AC at all. *c-Fos* expression is linked to the net increase of intracellular calcium (Sheng et al., 1993) triggered, for example, by opening of NMDA receptor Ca^2+^ channels in response to neuronal stimulation. This suggests that the pattern of graded intensity of FOS-labeling of cells as seen in the AC here (Figures 3A,B) is, first of all, the result of different Ca^2+^ influx in sound activated AC cells and, thus, comparable with the differences of transient Ca^2+^ levels in cells demonstrated by two-photon calcium imaging of sound stimulated mouse AC (Rothschild et al., 2010; Bathellier et al., 2012; Grienberger et al., 2012). The pattern of relatively few highly activated neurons detected both by FOS-labeling and Ca imaging is compatible with the concept of sparse coding in the AC (Hromádka et al., 2008; Sakata and Harris, 2009). The second mechanism of *c-Fos* expression is based on the increase of cAMP levels by neurotransmitters binding to G protein-coupled receptors (Sheng et al., 1990) so that activation e.g., of the cholinergic and dopaminergic systems in the course of changing attention, motivation, emotion, and task learning and performance (Missale et al., 1998; Picciotto et al., 2012) may lead to increased intracellular FOS levels. This mechanism of *c-Fos* expression comes into play with variables of the internal state, action in a certain behavioral context, or learning new stimuli and their contexts (Zhu et al., 1995; Herrera and Robertson, 1996; Kovács, 1998; Kandiel et al., 1999; Svarnik et al., 2005; Bourgeois et al., 2012). Both mechanisms leading to c-Fos expression are subject of influences by anesthetics such as the ketamine/xylazine anesthesia used in group G animals. We found a general reduction of FOS labeling in all AC fields except AI (Figures 5, 8), always most prominent in layers 2/3 (Figures 6, 8). Further experiments with other anesthetics have to show whether this pattern is specific for the type of anesthesia used.

In conclusion, we can expect state- and action-dependent differences between the awake animals of our experimental groups A–F to be expressed in the levels and patterns of FOS-labeling in the AC, which concerns both pyramidal cells and inhibitory interneurons (Staiger et al., 2002; Doron and Rosenblum, 2010; Gaykema et al., 2014).

### The message of the animals' behavior

Virgins with 1 day PE showed a high amount of non-maternal behavior (Figure 1). They retrieved pups only when they accidently hit them while moving around. The mothers, on the other hand, were focused on their routine in the context (Ehret, 2005), namely continuous, non-habituating phonotaxis and pup-retrieval, especially when hearing the ultrasound model (50 kHz long tones; group A) which they categorized without training as “attractive” in a choice test for categorical perception (Ehret, 1992). Group B mothers hearing the 50 kHz short tones, being non-preferred vs. the 50 kHz long tones in the choice test (Ehret, 1992), were not much disturbed in their maternal behavior (Figure 1), because mothers usually do a control run to the same place where they found a pup after having retrieved this pup, so that they found the next pup to be retrieved, etc. (Ehret and Haack, 1984). Thus, strange sounds are not obstructive to maternal performance of mother mice. The maternal performance of virgins with 5 days PE was intermediate between mothers and virgins with 1 day of PE (Figure 1). By 5 days of co-caring, these animals had implicitly (without training) learned to associate pups with ultrasounds, to pay attention to theses sounds and, as the mothers, prefer adequate ultrasound models (e.g., 50 kHz long tones) in choice tests against other sounds (Ehret et al., 1987; Ehret and Buckenmaier, 1994; Lin et al., 2013). Virgins with 5 days PE did not reach, however, the high level of maternal performance of mothers (Figure 1) because their maternal behavior was less focused. Increased levels of c-FOS in parts of the amygdala and ventromedial hypothalamus of virgins with 5 days PE (Geissler et al., 2013) suggest that they were somewhat stressed in the stimulus context for *c-Fos* expression. Low c-FOS levels in these parts of the limbic system in mothers and virgins with 1 day PE (Geissler et al., 2013) suggest little stress or excitement while hearing the sound for *c-Fos* expression.

In conclusion, our results suggest that mothers were hormonally primed to respond to the message of pup ultrasounds and did their instinct-based routine activated by the ultrasound. Virgins with 5 days PE had learned the message of pup ultrasounds but had not acquired the same level of routine as the mothers to act in a very focused way. Virgins with 1 day PE were still largely innocent of the message of the ultrasound and, therefore, their maternal behavior toward the pups was not focused at all.

### Activation of AI, AAF, and UF reflects integration of sound properties with animal state-dependent factors

A striking result of our study concerns the similarity between the groups of awake animals (A–F) in the numbers of highly activated cells all over AI, AAF, and UF both with regard to the total numbers of activated cells and their distribution across the cortical layers (Figures 3, 5, 6; IPI data). In the stimulus situation, all awake mice had in common to be permanently stimulated by sounds of changing location and by live mouse pups (odor, tactile), and be motor active most of the 15 min stimulation time. Being just motor active in retrieving pups who occasionally emitted ultrasounds (average 6 calls/min by 1 day old pups, 6–30 calls/min by 5 days old pup; Haack et al., 1983; Gaub et al., 2010) did not produce FOS labeling in the auditory cortex of mothers (Fichtel and Ehret, 1999). Therefore, the continuous presence of sounds, independent of the type of sound, together with motor activity and motivation are the conditions that must have caused the homogenous background activation level. Multisensory integration has been shown to modulate auditory processing in AI (Bizley et al., 2007; Lakatos et al., 2007; Kayser et al., 2009; Musacchia and Schroeder, 2009; Cohen et al., 2011; Scheich et al., 2011; Cohen and Mizrahi, 2015) and may contribute to the activation background. Previously, we studied with the same methods FOS-labeling in the mouse AC in response to pup wriggling-call models (3.8+7.6+11.4 kHz each at about 60.5 dB SPL) and found in awake mothers a background activation of average 5–7 cells/section in AI, AAF, and UF (Geissler and Ehret, 2004). This is nearly half the number of labeled cells in our present AI/AAF background (13.5 cells/section), and about the same number as in the anesthetized animal UF and AAF (Figure 5). In the wriggling call experiment, the mothers were quietly nursing their pups not leaving the nest during sound stimulation, a situation of motor inactivity comparable with that of anesthetized animals in the present study. Together, these observations suggest that tones of any frequency that are heard well above the perceptual threshold lead to activation of the whole AI, AAF, and UF, while the actual level of activation, here expressed by the number of highly activated cells, is dependent on the state of the animal, i.e., anesthetized, quietly awake, motor active. This hypothesis is supported by studies on human AC indicating a sustained sound-evoked response level that may or may not be modified by transient sounds or sounds to be attended (Linden et al., 1987; Bidet-Caulet et al., 2007), and further studies showing that ketamine anesthesia reduced spontaneous rates and sound-evoked activation in the cat AC (Zurita et al., 1994), slow-wave sleep reduced sound activation in the human AC (Czisch et al., 2002) and in most AC neurons of guinea pigs (Edeline et al., 2001), and background activity increased with increasing arousal in the cat AI during classical conditioning (Weinberger et al., 1984) and during motor task performance compared to passive listening in the monkey AI (Scott et al., 2007).

On top of the background labeling, wriggling call models, whether meaningful or not, led to peaks of FOS-positive cells at the tonotopically correct positions in AI and AAF of awake animals with an increment of average 6 cells/section (Geissler and Ehret, 2004). Similarly, an increment of average 6 cells/section was obtained with meaningful or non-meaningful 50 and 20 kHz models at the tonotopically correct positions in AI and AAF for 20 kHz (Stiebler et al., 1997; Joachimsthaler et al., 2014) or in the whole UF for 50 kHz (Figures 5, 6; present study). Further, extra-tonotopical activation peaks (increment of about 6 cells/section in the peak area) in response to 50 kHz occurred at the tonotopical position of 20 kHz (Stiebler et al., 1997; Joachimsthaler et al., 2014) in the middle of AI and AAF in awake animals (Figures 3, 5, 6). Since these peaks were restricted to cortical layers 2/3 (Figure 6), they may be explained by convergence of input to these layers through intracortical horizontal projections from layers 2/3 of the activated mouse UF (Hofstetter and Ehret, 1992) and thalamocortical input to neurons with characteristic frequencies near 20 kHz but broad tuning so that these neurons responded also to 50 kHz tones (Linden et al., 2003; Joachimsthaler et al., 2014). The thalamocortical input to neurons with characteristic frequencies near 20 kHz may also be the result of cochlear distortion products near 20 kHz produced by the ultrasounds and transmitted via cochlear nucleus (Roberts and Portfors, 2015) and inferior colliculus (Portfors et al., 2009) to the 20 kHz tonotopical position in the AI and AAF. Extra-tonotopical intracortical information transfer, here from UF to AI and AAF, has also been shown in the primary AC of other mammals (Budinger et al., 2000; Douglas and Martin, 2004; Metherate et al., 2005). Since the intracortical horizontal projections can be inhibitory or may exert strong inhibitory effects at their projection sites (Kurt et al., 2008; Happel et al., 2010; Moeller et al., 2010), the activation peak, similar as local electrical stimulation in layers 2/3 (Kubota et al., 1999) seems not to be forwarded to strongly activate the output layers 5 and 6 (Figure 6). This interpretation is compatible with results from electrophysiological recordings in awake mice showing that mouse pup ultrasounds excite neurons in the UF but exert substantial inhibition in neurons tuned to lower frequencies in the AC (Galindo-Leon et al., 2009).

In conclusion, our results support the initial hypothesis 2, and suggest UF as part of the primary AC (hypothesis 5). Unexpectedly and in mismatch of tonotopic representation, ultrasounds are processed locally in AI and AAF without significant activation, however, of cortical output layers 5/6 in all respective groups (A–E). In addition, the background activation across all layers in these primary fields relates to levels of arousal and motor activity of the animals reflecting their internal state (anesthetized, quietly awake, motor active), however only in the presence of sounds. Hence, hypothesis 6 is not confirmed for primary AC, strengthening hypothesis 2 not only for primary AC in general but also specifically for processing in the cortical layers.

### Activation of the higher-order field AII reflects the news value of a given sound in a certain behavioral context

Another striking result of our study concerns the group-specific activation in AII, separating mothers and virgins (Figure 8). Therefore, AII is a candidate for the application of hypothesis 3. The following discussion will show that activation of AII does not reflect just familiarity with a sound as previously suggested (Wan et al., 2001), and it does not reflect meaning either, but may express the “news value” of a given sound in a certain behavioral context. Mothers are adapted to the maternal context to which the ultrasounds (50 kHz long tones) belong as the regular signals. Therefore, 50 kHz long tones are the expected ones, and when they actually occurred they had little or no news value to mothers of group A. Mothers of group B perceived incoherence between the unexpected ultrasounds (50 kHz short tones) and the familiar context (deviant ultrasounds, familiar context) which produced a high news value, like an “aha effect.” Virgins of group C prefer 50 kHz long tones vs. 20 kHz long tones after 5 days co-caring experience with pups (Ehret et al., 1987; Ehret and Koch, 1989; Ehret and Buckenmaier, 1994), i.e., they learned to associate pup ultrasounds (the 50 kHz long tones) with the maternal context in order to adequately respond to the sounds. The learning success is still instable because the virgins appear to be stressed in the situation (Geissler et al., 2013). Hence, they are not yet in a maternal routine and not yet well adapted to expect the 50 kHz long tones as the regular signals that call for maternal responding. Therefore, 50 kHz long and short tones are assumed to have high news value in the maternal context for virgins of groups C and D in the sense that these tones either confirm and consolidate (50 kHz long tones, group C) or challenge (50 kHz short tones, group D) their present learning experience. Virgins of group E had the 1 day experience with pups and their ultrasounds and started to perceive series of 50 kHz long tones as adequate ultrasound models with news value. For group F virgins, the 20 kHz tones were unfamiliar, without consequences and without similarity with vocalizations of the species' repertoire and, thus, irrelevant or without news value. Similarly, the anesthetized females (group G) heard sounds independent of their context without any response option making the sounds irrelevant or without news value. Thus, the term “news value” on a scale from low to high characterizes the amount of information an animal perceived from a sound with certain acoustic properties in a given behavioral context in order to adjust its behavior. Now, with regard to the numbers of FOS-positive cells in AII (Figure 8), these numbers closely correlate with the assumed news value of the sounds, as explained. No or little news value produced little FOS labeling, even below the grand average background (groups A, F, G), higher FOS production relates to higher news value (groups C–E), and the highest number of FOS-positive cells, significantly above the average background level, is related to the highest news value (group B). This relationship between the amount of FOS labeling and the news value is supported by our previous studies: Incoherence between ultrasounds and context (familiar ultrasound, deviant context since the tones came from above the cage) produced high numbers of FOS-positive cells in AII of mothers (Fichtel and Ehret, 1999), and familiar or deviant pup wriggling calls in a familiar maternal context produced low or high numbers, respectively, of FOS-positive cells in AII (Geissler and Ehret, 2004).

AII activation demonstrates an important difference depending on how knowledge about coherence of ultrasound and context has been acquired (hypothesis 3). Hormonal and sensory priming during pregnancy and giving birth in the mothers (Rosenblatt et al., 1988; Numan and Insel, 2003; Scanlan et al., 2006; Brunton and Russell, 2008; Pinaud and Tremere, 2012) can be regarded as the basis for establishing traces of emotional memory in AII (Sacco and Sacchetti, 2010) that were matched in group A and mismatched in group B mothers. This does not mean that the high number of activated cells in group B correspond to the mismatch negativity recorded in human and animal AC to deviant sounds (Garrido et al., 2009; Fishman and Steinschneider, 2012; Jung et al., 2013) because in our paradigm the deviance was detected not in an ongoing stream of sounds but by comparison with stored memory. Retrieval of memory formed in a visuoauditory conditioning task involves high activation of the entorhinal cortex (Chen et al., 2013). High activation in the piriform cortex and the entorhinal cortex of virgins of groups C (Geissler et al., 2013) suggests that these virgins were consolidating and retrieving the memory established by implicit learning of the context, i.e., physical presence of pups, their ultrasounds and odor (Gottfried et al., 2004; Squire et al., 2004). The entorhinal cortex of the mothers, however, was not highly activated in the present context (Geissler et al., 2013) suggesting that the mothers did not retrieve memory via the entorhinal cortex. Together, these observations of the activation of the limbic system (Geissler et al., 2013) and AII (present study) suggest that AII activation according to the news value of a given sound is modulated differently during retrieval of primed contextual memory (no special involvement of the entorhinal cortex) in an instinct-based action compared to retrieval of memory established by implicit learning (entorhinal cortex involved). If mouse AII belongs as secondary AC to the anterior/ventral “what” pathway (Romanski et al., 1999; Kaas and Hackett, 2000; Malhotra and Lomber, 2007) and is analogous to monkey belt AC (Rauschecker et al., 1995) one would predict some selectivity of AII to species-specific sounds. Our studies indicate that this selectivity is evaluated in AII always relative to the actual behavioral context of sound perception and to the way contextual memory had been established.

### Activation of the higher-order field DP reflects the level of motivation and, by left-hemisphere advantage, the meaning of sounds in a context

DP activation shows a remarkable left-hemisphere advantage in the animals of groups A and C (Figure 8), i.e., in those who recognize the 50 kHz long tones as adequate models of pup ultrasound (Ehret, 1992; Ehret and Buckenmaier, 1994). It seems that the left-hemisphere dominant recognition of adequate models of mouse pup ultrasound by the mothers (Ehret, 1987) is reflected by the strong left-right difference in activation of DP here (Figure 8). Virgins after being explicitly trained in an operant-reward conditioning paradigm to prefer 50 kHz long tones did not show the left-hemisphere advantage of 50 kHz recognition (Ehret, 1987). In those tests, the virgins were in a two-alternative choice situation in which they could use working memory to decide for the correct tone. Here, they had to recall stored memory information in order to estimate whether the perceived ultrasound belonged to the same category as the ultrasounds they had associated with pup presence during the period of 5 days social experience. Combining these data and the left-hemisphere dominant activation of DP while perceiving acoustically adequate (not inadequate) wriggling calls (Geissler and Ehret, 2004), we propose that enhanced left-hemisphere processing in DP reflects call recognition or the perception of the meaning of communication sounds in a given behavioral context. Clearly, the condition for enhanced left-hemisphere processing in DP of mothers and experienced virgin females includes the presence of acoustically adequate sounds in a compatible behavioral context and active animals (hypothesis 4). Different from AII activation, the left-hemisphere dominant activation of DP seems to be independent of whether the match between sound and context was established via primed contextual memory or memory based on implicit learning.

Maternal motivation, i.e., the tendency to behave maternally, is also correlated to the number of highly active cells in DP (hypothesis 4). Anesthetized animals (group G) were not motivated at all resulting in a low number, below the grand average background, of FOS-positive cells (Figure 8). Virgins with 1 day PE with low maternal motivation (Figure 1) had significantly higher numbers, equaling the average background. Virgins with 5 days PE (group D) and mothers of group B were even more motivated (Figure 1) and, when listening to the inadequate ultrasound model, had the same significantly increased numbers of FOS-positive cells in both hemispheres (Figure 8). Interestingly, the sum of the average numbers of FOS-positive cells from both hemispheres of group B and D equals exactly the sum of the average numbers of FOS-positive cells from both hemispheres of group A and C (Figure 8). It seems that motivation provides the same background activation of DP in both hemispheres and, in the case of adequate sound pattern for the perception of meaning (groups A, C), this activation is enhanced in the left and suppressed in the right hemisphere. Left- over right-hemisphere enhanced activation in response to pup ultrasounds has recently been shown via cell-attached recordings in the AI of house mouse mothers and virgin females with at least 3 days PE but not in inexperienced virgins (Marlin et al., 2015). Since we did not see significant hemisphere differences of activation in the AI of any experimental group (Figures 5, 6), we suggest that at the input-side to AI neurons, the activity of which is characterized by the FOS-labeling intensity, the left-hemisphere enhancement was not yet present, at least not detectable with our method. Because it was present in the spiking activity (spike rate and spike timing precision; (Marlin et al., 2015)) characterizing AI output, left hemisphere advantage seems to be added during processing of acoustically adequate ultrasound in AI. Inadequate ultrasound and other sounds have not been tested. The introduction of the left-hemisphere advantage in processing (at least of meaningful sounds) may result from a left-hemisphere advantage of oxytocin-receptor expression and oxytocin action in AI, both enhancing neuronal activation and the association of ultrasounds with other pup cues (Marlin et al., 2015). Unfortunately oxytocin-receptor distribution and oxytocin action has not yet been studied in other auditory cortical fields besides AI, so that we can only speculate about how oxytocin may influence left-hemisphere dominant activation in DP. The oxytocin data (Marlin et al., 2015) suggest that UF and AI input to DP (Hofstetter and Ehret, 1992) are more intense and temporally more precise on the left compared to the right DP of mothers and virgins with 5 days PE, and not different from the right DP in virgins with only 1 day PE, explaining at least part of the laterality of DP activation in our study. Further evidence about oxytocin action in mice and humans has shown lowering of the threshold for initiating maternal behavior (Rich et al., 2014), high oxytocin blood levels during parturition (Douglas et al., 2002), and increased oxytocin blood levels after tactile contact with young (Feldman et al., 2010; Nagasawa et al., 2012). Taking together this evidence and the data from Marlin et al. (2015) and from our present study, we arrive at the conclusion that oxytocin appears as a key player (a) in priming the mothers for being maternal right after parturition by activating innate releasing mechanisms for responding to pup cues and/or by facilitating rapid learning of pup cues, (b) in enhancing the learning of virgins to behave maternally, and (c) in controlling the left AC to respond to sounds that call for maternal care.

Perception of the meaning of ultrasounds by adult mice in the pup-caring context initiates adequate action, i.e., orienting toward the sound source (Ehret, 2005). Directed orientation behavior may especially be supported by the DP because of its dorsoposterior position in AC that suggests DP as part of the “where” or “how” pathway to the frontal cortex (Romanski et al., 1999; Kaas and Hackett, 2000; Malhotra and Lomber, 2007). Similarly, a comprehensive approach to the cortical organization of human speech processing assumes that the dorsal stream from recognition to action is strongly left-hemisphere dominant, while the ventral stream is largely bilaterally organized (Hickok and Poeppel, 2007) as seen in the activation of the mouse AII (Figure 8). In addition, posterior (secondary) AC in humans has a left-hemisphere activation advantage for categorization of sounds in the time domain (Brechmann and Scheich, 2005; Gourévitch et al., 2008) and of series of natural laughing and crying vs. backward laughing and crying (Sander and Scheich, 2005). These examples of lateralization in human AC are comparable with the categorizations of ultrasounds by the mice which are also based on memory about series of sounds with a critical time domain parameter (Ehret, 1992) followed by the initiation of adequate action. Therefore, common neural substrates and networks for lateralization in human and mouse secondary (higher-order) AC (DP in the mouse) seem to exist that may be basic to the recognition not only of speech sounds but also of communication sounds in general. This conclusion is in accordance with hypothesis 7.

### Left-hemisphere advantage of AC functional size

The left AC of all experimental groups was activated over a significantly larger area than the right AC supporting hypothesis 1 (Figure 4). The left-side advantage with a size factor of 1.11 rostrocaudally and 1.06 dorsoventrally equals the functional left-right size relationships previously found in the mouse AC (Stiebler et al., 1997; Geissler and Ehret, 2004). In most humans, the planum temporale is larger on the left compared to the right side (Galaburda et al., 1978; Penhune et al., 1996; Shapleske et al., 1999). This seems to be true also for primates (Gannon et al., 1998) and, as our mouse data show, may be a more general feature of the AC of mammals. Since the activated AC size on the left hemisphere is larger than on the right side not only in awake but also in anesthetized animals (Stiebler et al., 1997; Figure 4, present study), attention to sound stimuli is not a necessary condition for the increased size of sound-activated brain area on the left side. Functional left-hemisphere advantage of sound-evoked activation has been found below the cortex in the thalamus (Mateer and Ojemann, 1983; Hugdahl et al., 1990; King et al., 1999) and even in otoacoustic emissions of the cochlea (Sininger and Cone-Wesson, 2004) suggesting that it represents a rather general phenomenon of the auditory system for processing acoustically complex, especially time-critical information from the inner ear to perception and recognition (Ehret, 2006).

## Author contributions

GE conceived the work. GE and DG designed the study. DG and SS performed the experiments and analyzed the data. GE and DG wrote and SS revised the paper. GE, DG, and SS approved the version to be published.

### Conflict of interest statement

The authors declare that the research was conducted in the absence of any commercial or financial relationships that could be construed as a potential conflict of interest.
